# Cerebral Diplegia Following Birth Injury
*This paper formed the substance of a short address illustrated by lantern slides given before the Bristol Medico-Chirurgical Society on 12th May, 1948.


**Published:** 1948

**Authors:** R. M. Norman

**Affiliations:** Medical Superintendent, Stoke Park Colony, Bristol. Special Lecturer in Neuropathology, University of Bristol


The Bristol
Medico-Chirurgical Journal

*" Scire est nescire, nisi id me
Scire alius sciret."*

SUMMER, 1948.

### CEREBRAL DIPLEGIA FOLLOWING BIRTH INJURY*

BY

R. M. Norman, M.D.

*Medical Superintendent, Stoke Park Colony, Bristol.*
*Special Lecturer in Neuropathology, University of Bristol.*

The subject of cerebral palsy is attracting renewed interest in this country owing to the formation of a British Council for the Welfare of Spastics and the setting up at Croydon and elsewhere of special treatment centres for spastic and athetoid children. Mainly as a consequence of the work of Phelps in America it is becoming more generally realized that well-devised training methods may bring about substantial improvement in those diplegics who retain a fair degree of intelligence. There is also reason to believe that the number of such handicapped children in the community is far from negligible, since in Bristol Dr. Smallwood has discovered seventy cases of cerebral palsy in a school population of fifty thousand. It would seem, then, that the social implications of cerebral palsy are sufficiently important to justify further research : for while it may be said that advances have been made in treatment, at any rate for the fortunate few, there still remains much to be discovered as regards the causation and perhaps the prevention of this common form of paralysis in childhood. In particular, the part played by birth-injury in the pathogenesis of the condition needs to be more fully investigated.

Since the time of W. J. Little's classical paper to the Obstetrical Society of London in 1861 an association between cerebral palsy

* This paper formed the substance of a short address illustrated by lantern slides given before the Bristol Medico-Chirurgical Society on 12th May, 1948.

and abnormal circumstances of birth has often been shown to exist. Bridgeman, for example, in 1942, found that in a group of 113 spastic children histories of abnormal presentation, premature birth and prolonged labour were many times more frequent than in a control series of normal babies born in a maternity hospital. Even more significant was the frequency among the spastics of convulsions, cyanosis and abnormal limpness or stiffness of the limbs during the early neo-natal period. More than half the spastics were first-born and no familial incidence was found in the whole group, which included seventy-eight diplegics.

Despite findings of this sort there is still a strange reluctance on the part of neurologists, or at any rate of those who write textbooks, to admit that cerebral diplegia may arise from injury to the brain at birth. This scepticism probably reflects the influence of the late James Collier who, in his presidential address to the Royal Society of Medicine in 1923, strongly opposed the theory, then widely held, that meningeal hæmorrhage at birth led to atrophy of the underlying convolutions. Since that time, however, the pathology of birth-injury has been more thoroughly studied, and it is now known that those lesions likely to cause bilateral paralysis occur within the substance of the brain and are not related to hæmorrhage over its surface.

Brief reference must be made at this point to the influential work of Schwartz (1924) and his associates who attributed these intracerebral bleedings and softenings in the new-born to the consquences of extreme engorgement of the cerebral venous systems. According to this school of thought the cortical lesions of birth-injury are related to the veins draining into the superior longitudinal sinus, while lesions of the basal ganglia and central white matter of the hemispheres follow circulatory disturbances in the drainage area of the great vein of Galen, a vessel notoriously liable to damage during the process of birth. Apart from the destructive effect of gross hæmorrhage from congested veins, which is especially apt to occur in premature infants, extensive and apparently progressive softening of the brain may follow severe vasodilatation and circulatory stasis in the smaller venules and their capillaries (Marburg and Casamajor, 1944).

The following account of the clinical and pathological findings in cases of diplegia attributable to birth-injury is based upon the study of nine cases in which a detailed examination of the brain has been made.

*Birth histories.*—Of these nine cases all were first-born and eight were males. There were two twin births in the series. Low birth-weights ($4\frac{1}{2}$ to $5\frac{1}{2}$ lb.) were recorded in four instances, the birth being very rapid (under two hours) in two of these and precipitate in one. Dry labour with a post-mature infant was recorded once and in another case pelvic contracture in the mother was known to have been present. Severe asphyxia was a feature of three cases and in two others there was apathy with inability to take the breast. Three infants showed twitching of the limbs during the first few days of life and, in two, recurrent attacks of cyanosis were observed. Subsequent epilepsy developed in six cases.

*Clinical types.*—Six patients showed during life a symmetrical spastic paresis of the limbs with contracture of hamstrings and adductors and the usual signs of upper motor neurone deficiency. In two cases the paralysis was unequally distributed on the two sides of the body, a hemiplegia with contractures being combined with a less severe spastic paresis of the other leg. Rigidity of extra-pyramidal type with associated athetosis was present in one case.

*Pathological findings.*—As Benda (1945) has recently demonstrated, the late neuropathological sequelæ of birth-injury are very diverse and vary from a diffuse loss of nerve-cells to an almost complete destruction of the cortex of both hemispheres. The following lesions may be found singly or in conjunction with one another in brains of diplegic patients :

1. Lesions of the central white matter and basal ganglia (drainage area of the Great Vein of Galen) :

(*a*) Softening of the central white matter of the hemisphere, often amounting to actual cavitation, may be present in the tissue adjacent to the outer angle of the lateral ventricle and may extend throughout the brain from almost the frontal to the occipital pole. This lesion, as Schwartz has shown, is very characteristic of birth-injury and when present on both sides leads to idiocy. The pyramidal tracts remain at about the stage of development found in the newly-born infant and as time passes the typical picture of Little's disease with scissor posture of the legs becomes manifest.

(*b*) *État marbré of the corpus striatum* is characterized by small areas of nerve-cell loss associated with peculiar networks of abnormally dense myelinated fibres. The clinical picture is that of athetosis, and when the destructive process is confined to the striatum the intelligence of the patient may remain high. The same condition may also be present in the thalamus. Other extra-pyramidal syndromes may follow destructive lesions of the basal ganglia in cases of birth-injury.

2. Cortical lesions.

(*a*) "*Atrophic sclerosis*" (*ulegyria*). In this condition certain convolutions appear shrunken and tough to the touch on account of the neuroglial overgrowth which is secondary to nerve-cell destruction. In the variety of ulegyria associated with birth-injury the destructive process tends to vary sharply in its local intensity. Thus the basal parts of affected gyri are frequently involved while the crowns may escape, or sometimes one-half only of the convolution is destroyed. In the adjacent preserved grey matter of the cortex one may often see an overgrowth of myelinated fibres (état marbré of the cerebral cortex). Adjoining gyri often show a diffuse thinning of the nerve cells in the outer part of the cortex, the third or pyramidal cell-layer being the most affected.

(*b*) *Multiple cortical cavitations* ("*poly-porencephaly*"). The cortex may be destroyed over a wide area by the formation of numerous small cavities involving the subcortex and the central cores of white matter of the individual gyri. Superficially thecontour of each convolution is recognizable, but the cortical tissue is reduced to a thin rind representing the outer or molecular layer.

(*c*) *Laminar atrophy.* Sometimes in convolutions normal to the naked eye microscopical examination reveals a thin streak of sharply defined nerve-cell loss in the mid-zone of the grey matter. This laminar atrophy is usually represented in neuroglial preparations by an equally clear-cut streak of fibrillary gliosis, indicating that the condition is the result of destruction rather than of malformation. Such lesions, though of limited extent, when placed in the upper part of the motor cortex are sufficient to lead to a clumsiness of the contra-lateral leg associated with a positive Babinski reflex. This type of minimal pathological lesion may very probably underlie those clinical types of spastic paresis in which the intelligence remains relatively intact.

3. *Massive softening of the white matter combined with cortical atrophy.*—In this rare condition, represented by one case in this series, a thin shell of gliotic cortex forms the outer wall of a large cystic cavity lying in the substance of the hemisphere external to the ependymal lining of the ventricle. Fine strands of preserved white matter pass in a trabeculated network between these two limiting walls. According to Marburg and Casamajor (1944) this condition of " central porencephaly " is the consequence of a process of progressive encephalomalacia which may be initiated by birth-injury. In my own case, that of an infant dying at the age of eighteen months, sclerotic lesions of considerable age were found lying side by side with those of more recent origin, the latter consisting of large foci of densely packed phagocytic microglial cells containing lipoid.

### SUMMARY

An account has been given of the neuropathological features presented by nine cases of cerebral diplegia in each of which the birth history was abnormal or suggestive of intra-cranial damage. These lesions, which are presumed to be late sequelæ of birth injury, fall into two main groups, those affecting the cerebral cortex being attributable to hæmorrhage from or circulatory stasis in veins draining into the superior longtiudinal sinus, while the often symmetrically placed lesions of the central white matter of the brain and basal ganglia are referable to similar vascular disturbances in areas drained by the tributaries of the great vein of Galen.

### REFERENCES

Benda, C. E., 1945. *Medicine*, **24,** 71.
Bridgeman, O., 1942. *Am. J. Dis. Child.* **64,** 11.
Collier, J. S., 1924. *Brain*, **47,** 1.
Marburg, O., and Casamajor, L., 1944. *Arch. Neurol Psychiat.* (Chicago), **52,** 170.
Schwartz, Ph., 1924. *Z. ges. Neurol. Psychiat.* **90,** 263.

